# The Impact of Amorphisation and Spheronization Techniques on the Improved *in Vitro* & *in Vivo* Performance of Glimepiride Tablets

**DOI:** 10.15171/apb.2017.067

**Published:** 2017-12-31

**Authors:** Rana Refaat Makar, Randa Latif, Ehab Ahmed Hosni, Omaima Naim El Gazayerly

**Affiliations:** ^1^Faculty of Pharmacy, Ahram Canadian University, Egypt.; ^2^Faculty of Pharmacy, Department of Pharmaceutics, Cairo University, Cairo, Egypt.; ^3^Faculty of Pharmacy, Russian University, Egypt.

**Keywords:** Dissolution, Pharmacodynamic study, Blood glucose level, Matrix tablets, Spherical agglomeration, Triple solid dispersion adsorbate

## Abstract

***Purpose:*** Triple solid dispersion adsorbates (TSDads) and spherical agglomerates (SA) present new techniques that extensively enhance dissolution of poorly soluble drugs. The aim of the present study is to hasten the onset of hypoglycemic effect of glimepiride through enhancing its rate of release from tablet formulation prepared from either technique.

***Methods:*** Drug release from TSDads or SA tablets with different added excipients was explored. Scanning electron microscopy (SEM) and effect of compression on dissolution were illustrated. Pharmacodynamic evaluation was performed on optimized tablets.

***Results:*** TSDads & SA tablets with Cross Povidone showed least disintegration times of 1.48 and 0.5 min. respectively. Kinetics of drug release recorded least half-lives (54.13 and 59.83min for both techniques respectively). Cross section in tablets displayed an organized interconnected matrix under SEM, accounting for the rapid access of dissolution media to the tablet core. Components of tablets filled into capsules showed a similar release profile to that of tablets after compression as indicated by similarity factor. The onset time of maximum reduction in blood glucose in male albino rabbits was hastened to 2h instead of 3h for commercial tablets.

***Conclusion:*** After optimization of tablet excipients that interacted differently with respect to their effect on drug release, we could conclude that both amorphisation and spheronization were equally successful in promoting in vitro dissolution enhancement as well as providing a more rapid onset time for drug action in vivo.

## Introduction

A variety of technical problems are usually encountered in the pharmaceutical industry when dealing with the formulation of insoluble drugs,^1^ often leading to a suboptimal drug product. Poor aqueous solubility of drugs affects both their *in vitro* dissolution rate,^2^ as well as their pharmacological activity.^3^ Therefore, continuous efforts have been dedicated for the treatment of such problem through a different design of particle technology^4^ and particle engineering processes, such as spray freezing into liquids,^5^ sonocrystallization,^6,7^ and others.^8^ In general, most preliminary pretreatment of particles relies upon making a change in the drug crystallinity, the so-called amorphisation techniques.^9^ Amorphous forms of drugs are characterized by a disordered arrangement of molecules in the solid state. This is accompanied by a higher state of free energy, enabling faster extent and rate of drug dissolution.^10,11^ Another well-known strategy for decreasing drug crystallinity is particle spheronization which was achieved in literature via different techniques and mechanisms;^12^ thus, enabling dissolution enhancement of poorly soluble drugs.^13^

Direct tabletting of pharmaceutical materials involves dry blending and compaction of the active pharmaceutical ingredient with the necessary excipients and lubricants. The whole process is simple and saves time, costs and energy.^14,15^

Many excipients were found helpful in the design of a proper formulation when they were incorporated during tablet manufacture.

In some cases, the addition of diluents might contribute to enhancing the dissolution of poorly soluble drugs.^16^ Lactose is one of the most famous diluents used in pharmaceutical formulation. It gained much popularity due to its good physical properties, being pleasant in taste, non hygroscopic, readily soluble in water and non-reactive with most excipients.^17^ Khan and Zhu^18^ revealed that tabletting with lactose resulted in a limited enhancement in the release rate of ibuprofen. Lin^19^ also found an increase in the release rate of theophylline from tabletted microcapsules containing lactose. Mannitol (Pearlitol SD) was selected in some formulae as diluent owing to its low hygroscopicity and good flowability. Gonnissen et al.^20^ believed that mannitol imparted an acceptable tensile strength to the tablets.

It was also shown that many binders had a very good influence on the dissolution profile of drugs. Chitosan, when used as a binder, affected the mechanical properties of granules, the disintegration time of tablets, and the whole dissolution profile of chlorpheniramine maleate was enhanced.^21^ Avicel PH 102 as a direct compression excipient^22^ produced tablets with lower crushing strength, shorter disintegration time and smaller weight variation as compared to Avicel PH101.^23^ Low substituted hydroxypropyl cellulose (L-HPC) had also good binding and disintegrating properties when used in fast disintegrating tablets along with Avicel PH102.^24^ Moreover, increased amount of L-HPC in the prepared granules of sparfloxacin resulted in increasing its dissolution rate. The polymer induced a considerable expansion in the matrix of the film-coated granules due to their uptake of water from the dissolution medium. The process resulted in film bursting after a short lag time.^25^

Many superdisintegrants were found to be successful in tablet formulations.^26,27^ Generally, starch disintegrants tended to swell and disrupt the tablet or helped disintegration by particle-to-particle repulsion.^17^ The pregelatinization process involved physical modification of the starch resulting in the combined benefits of the soluble and insoluble functions of starch. Its high swelling power could be achieved when hydrated with cold water. This produced viscous slurries that might have resulted in better wetting of drug matrices inside tablets.^28^ Ac-Di-Sol, a well known superdisintegrant, swelled 4-9 times its original volume when it came in direct contact with water. This helped water uptake by the tablet, causing its rapid breakage. The individual fibers of Ac-Di-Sol acted as hydrophilic channels to absorb and transfer water into the tablet system, giving rapid solubilization of tablet constituents and a higher disintegration and dissolution rate.^29^

Cross Povidone (CP) is a water insoluble polymer. Its particles possessed a porous morphology that initiated rapid water absorption and volume expansion. A probable hydrostatic pressure was then exerted on tablets, causing their disintegration.^30,20^

Generally, drugs may be incorporated inside tablets as simple powder^31,32^ or preformulated in other forms. Solid dispersion of poorly soluble drugs prepared by several techniques were compressed into tablets in order to attain an enhancement in dissolution profiles of such drugs.^33-35^ Spherical crystals of several drugs were also compressed in the form of tablets. A considerable increase in the rate and extent of drug release from such formulae was illustrated.^36-38^

Trial for dissolution enhancement of glimepiride was achieved through the preparation of solid dispersion with either sodium starch glycolate^39^ or with PVPK30.^40^ However, to our knowledge; literature available on glimepiride lacks research study on spheronization or surfactant-aided solid dispersion.

The present study aims to test and compare the applicability of new amorphisation and spheronization techniques viz: Triple solid dispersion adsorbate (TSDads) or spherical agglomerates (SA) in attaining best results in dissolution enhancement of glimepiride, as well as studying the effect of compression on dissolution parameters. The work will involve an *in vitro* optimization of the tabletting process in the presence of different partially water-soluble to water-insoluble excipients. A pharmacodynamic evaluation is carried out on optimized formulae to test for the hastening in the onset of hypoglycemic action after oral administration compared to a marketed product.

## Materials and Methods

### Materials

Glimepiride was kindly supplied by Sedico Pharmaceuticals, Giza, Egypt. Sodium Lauryl Sulphate (SLS) was purchased from El-Nasr Pharmaceutical Chemicals Co., Cairo, Egypt. Pregelatinized starch (PreGelSt) was a gift from Colorcon Limited, UK. Ac-Di-Sol (Crosscarmellose sodium) was purchased from E. Merck, Germany. Crosspovidone XL (CP) and Avicel pH 102 were purchased from FMC Corporation, Philadelphia, USA. Starlac (lactose and maize starch), Pearlitol SD (Mannitol) and Pearlitol flash (mannitol and maize starch) were a gift from Roquette, France. Gelucire 50/13 was obtained from Gattefosé, France. Colloidal Silicon dioxide (Aerosil 200) hydrophilic was obtained from Degussa, USA. Polyvinylpyrrolidone (PVP K30) was obtained from Fluka, Switzerland. Low substituted Hydroxypropylcellulose (L-HPC) was purchased from Shin-Etsu Chemical Co., Ltd Tokyo, Japan. Spray-dried lactose was a gift from Ph. Francaise Co., France. Carbon tetrachloride and magnesium stearate were obtained from El-Nasr Pharmaceutical Chemicals Company, Cairo, Egypt. Aspartame was purchased from Sigma, St.Louis, USA. Amaryl® tablets (3 mg): Batch No. 2EG008 was obtained from Sanofi-Aventis, Cairo, Egypt.

### Methods

#### Preparation of ternary solid dispersion (TSD)

Glimepiride TSD was prepared with PreGelSt as a carrier by the melting method using Gelucire 50/13 as surfactant at a drug-to-carrier-to-surfactant ratio of 1:5:15, respectively. The drug and carrier were added consecutively with continuous stirring in the molten Gelucire until a homogenous dispersion was obtained. The mixture was then allowed to cool on an ice bath until solidification.

#### Preparation of ternary solid dispersion adsorbates (TSDads)

The melt adsorption technique described by Parmar et al.^41^ was used to prepare TSDads. In brief TSD was dropped (while in the molten state) onto lactose powder (preheated to 70 °C) with continuous stirring to obtain the respective TSDads at a drug-to-carrier-to-surfactant-to-adsorbent ratio of 1:5:15:30, respectively. The mixture was allowed to cool to room temperature where it continued to have the appearance of free flowing powder.

#### Evaluation and characterization of TSD &TSDads

Drug content uniformity: To test for homogeneity of drug content within batches of TSD & their adsorbates, ten random samples were taken from each batch. A fixed weight was stirred in methanol for 15 min, filtered and assayed spectrophotometrically for glimepiride content. Each experiment was done in triplicates.

Scanning electron microscopy: The surface morphology of glimepiride and formulae based on solid dispersion with the drug were visualized by scanning electron microscopy (SEM JSM-6390 LV, JEOL, Tokyo, Japan) at a working distance of 20 mm and an accelerated voltage of 15 kV. Samples were gold-coated with a sputtercoater (Desk V, Denton Vacuum, NJ, USA) before SEM observation under high vacuum of 45 mTorr and high voltage of 30 mV.

#### Preparation of spherical agglomerates (SA)

SA were prepared by a slight modification to the quasi-emulsion^42^ and crystallo-co-agglomeration.^43,44^ Glimepiride (150mg) was dissolved in 2ml dimethyl formamide at 25°C. To this solution were added Aerosil 200 (150mg) as dispersing agent^45^ and Starlac (0.5% w/v) as carrier^46^ with continuous agitation using a three-blade mechanical stirrer at 500 rpm to keep the suspension uniformly dispersed. PVP K30 was dissolved in water (4 ml) until a saturated solution (data not shown) was formed at room temperature, then the prepared aqueous solution, acting as poor solvent for the drug, was added to the drug solution with continuous agitation in order to precipitate the drug. Carbon tetrachloride (0.85ml), acting as a bridging liquid, was added drop-wise to the agitated dispersion. Formed agglomerates were collected after a further 10-minute agitation, washed with distilled water, filtered, dried in a hot air oven at 45°C for 24h and stored in tightly closed containers in a desiccator for further investigations.

#### Evaluation and characterization of SA

Drug content uniformity: Glimepiride content was tested within batches of SA. Ten random samples were taken from each batch. Spherical agglomerates were crushed in a glass mortar. A fixed weight was then stirred in methanol for 15 min, filtered and assayed spectrophotometrically for glimepiride. Each experiment was done in triplicates.

Scanning electron microscopy: Surface topography of glimepiride particles, pure excipients and prepared SAs were observed and compared through a scanning electron microscope (Joel Corp., Mikaka, Japan) operated at 15 Kv after coating with gold. Different magnification powers were illustrated.

#### Formulation of tablets 

Previously prepared TSDads and SA were compressed into tablets. Some superdisintegrants viz: PreGelSt, Starlac, Ac-Di-Sol, CP and Pearlitol flash were tried during compression. All added excipients were mixed with previously prepared TSDads or SA by the geometric dilution method; lactose was added as a diluent to adjust the final weight of the tablet to 250 mg. Powder mixtures was compressed using a single punch tablet press (Korsch EKO, Germany) using 6mm flat level edged punch. A compression force of (3-5 KN) was applied so as to provide a constant value for hardness for all tested formulae, and measured with tablet hardness tester (Coplay scientific type TH3/500 Nottingham, United Kingdom NG42J).

#### Evaluation of prepared tablets containing TSDads or SA

Prepared tablets were subjected to quality control tests following USP Pharmacopeial regulations, namely: weight variation,^47^ friability,^48^ and content uniformity.

Disintegration time (D.T.): The D.T. for six tablets from each formula was determined in distilled water at 37°C using USP disintegration tester (Coplay Scientific, NE4-COP, UK). The initial disintegration time (I.D.T.) was recorded at the beginning of disintegration. The time at which complete tablet disintegration occurred was recorded as total disintegration time (T.D.T.).

*In vitro* drug release: The release profile of the drug from prepared formulae was determined using USP dissolution tester (Hanson Research, 64-705-045, USA) type I at 100 rpm. Release was carried out at 37°C in 900ml 0.5% aqueous solution of SLS. Two ml samples were withdrawn at different time intervals and replaced with fresh media. Absorbance of the samples was measured spectrophotometrically at λ _max_ 228nm_._ Results were mean of three determinations.

Kinetic analysis of release data: Data obtained from release experiments were treated statistically according to linear regression analysis. Data were fitted to zero order, first order and Higushi diffusion model.

Equation for zero order: C=C°-K° t

Equation for first order:logC=logC°- Kt/2.303

Simplified equation for Higuchi diffusion model:

Q=K× t^1/2^

#### Physicochemical characterization of optimized tablet formulae containing TSDads or SA

Tablet formulae with TSDads or SA showing best results with respect to DT and dissolution profile were selected for further characterization.

Scanning Electron Microscopy (SEM): The surface topography and cross section of optimum tablet formulae T5 and TS2 were observed through a scanning electron microscope (Joel Corp., Mikaka, Japan) operated at 15 kv after coating with gold.

Effect of compression on glimepiride release: The components of tablet formulae T5 and TS2 were filled in hard gelatin capsules size 1 and subjected to release study under the same conditions as their respective tablets. Kinetic treatment of drug release data was then matched with results obtained from their respective tablets.

#### Pharmacodynamic evaluation of optimized tablet formulae

Optimized tablet formulae with the least recorded release t_1/2_ were further evaluated with respect to their pharmacodynamic effect on male albino rabbits.

The study protocol was approved by the institutional review board of the Faculty of Pharmacy, Cairo University (PI 1144).

The study was based on single dose and parallel group design. Male albino rabbits weighing 3.5-4kg were kept on standard diet and then made to fast overnight before carrying the experiment. They were divided into three groups, each of eight animals. Groups I, II and III for administration of marketed product Amaryl®, TS2 and T5 tablets respectively. All tested tablets contained an amount equivalent to 3 mg glimepiride. Blood samples after oral intake of glimepiride were withdrawn from the marginal ear vein of rabbits at specific time intervals; every 15 min. during the first hour, every 30 min. up to 3h, and then every hour up to 12h. Samples were measured for blood glucose level (BGL) using *ACCU CHEK® Go* system.^49,50^ Initial BGL was measured at zero time (just before the administration of the respective tablets). Each animal was considered as its own control and the hypoglycemic response was calculated as the percent reduction in blood glucose level according to the following equation

$$\text{Decrease BGL }\left(\text{\%}\right)\;=\frac{BGL\;at\;t=0-BGL\;at\;t=t\times 100}{BGL\;at\;t=0}$$

Mean percent reduction in BGL versus time was drawn and the area under the Curve (AUC _0-12_) was calculated adopting the trapezoidal rule.^51^ Maximum reduction (Red max) was attained in BGL and the time to reach Red max was denoted as Tmax was compared for both formulae and the marketed product. Statistical analysis of the results was performed using one-way analysis of variance (ANOVA) to determine the least significant difference between tested formulae.

## Results and Discussion

### Drug content evaluation in TSD, TSDads & SA

All assayed samples of TSD & TSDads resulted in 98-100% glimepiride content, indicating uniformity of drug distribution within different matrices. Samples of SA gave around 97-99% glimepiride content, indicating the absence of drug loss during the dispensing procedures.

### Scanning electron microscopy for TSD, TSDads & SA

**Figure 1:**
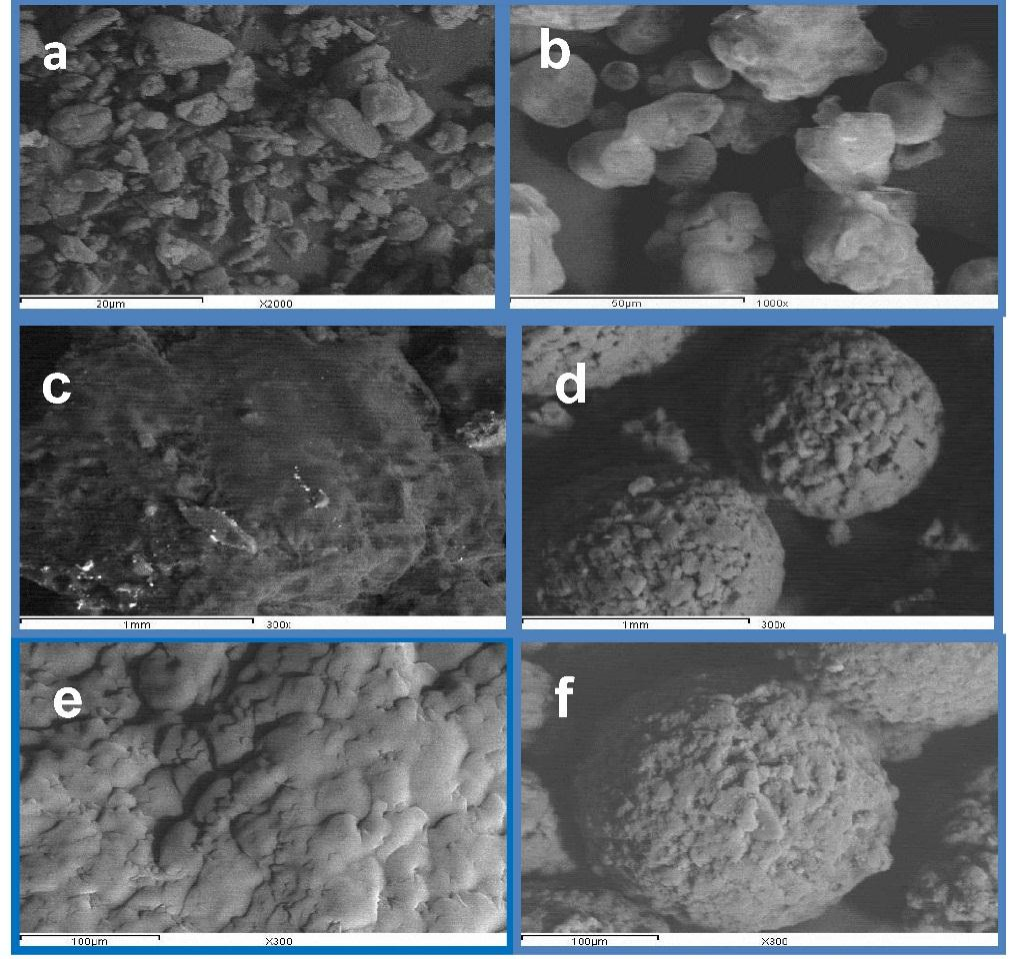
SEM of (a) glimepiride particles; (b) pregelatinized starch [PreGelSt]; (c) gelucire 50/13 ; (d) lactose; (e) triple solid dispersion[TSD] ; (f) triple solid dispersion adsorbate [TSD ads]

Figure1 shows the strong crystal habit of glimepiride platelets with distinct sharp edges and the gradual transformation that occurred into an amorphous structure with smooth to round edges through the formulation of TSD & TSDads. The surface of TSD acquired an amorphous shape with smooth texture similar to the surface topography of intact gelucire pellets. This obviously demonstrated the contribution of gelucire in the final amorphisation of the triple dispersion. TSDads showed a perfect spherical morphology with complete rounded edges coinciding to the surface structure of lactose. It could be, thus, clearly identified that the role of the adsorbent was not only restricted to disaggregation and micronization of particles, but also to promoting their spheronization.

**Figure 2:**
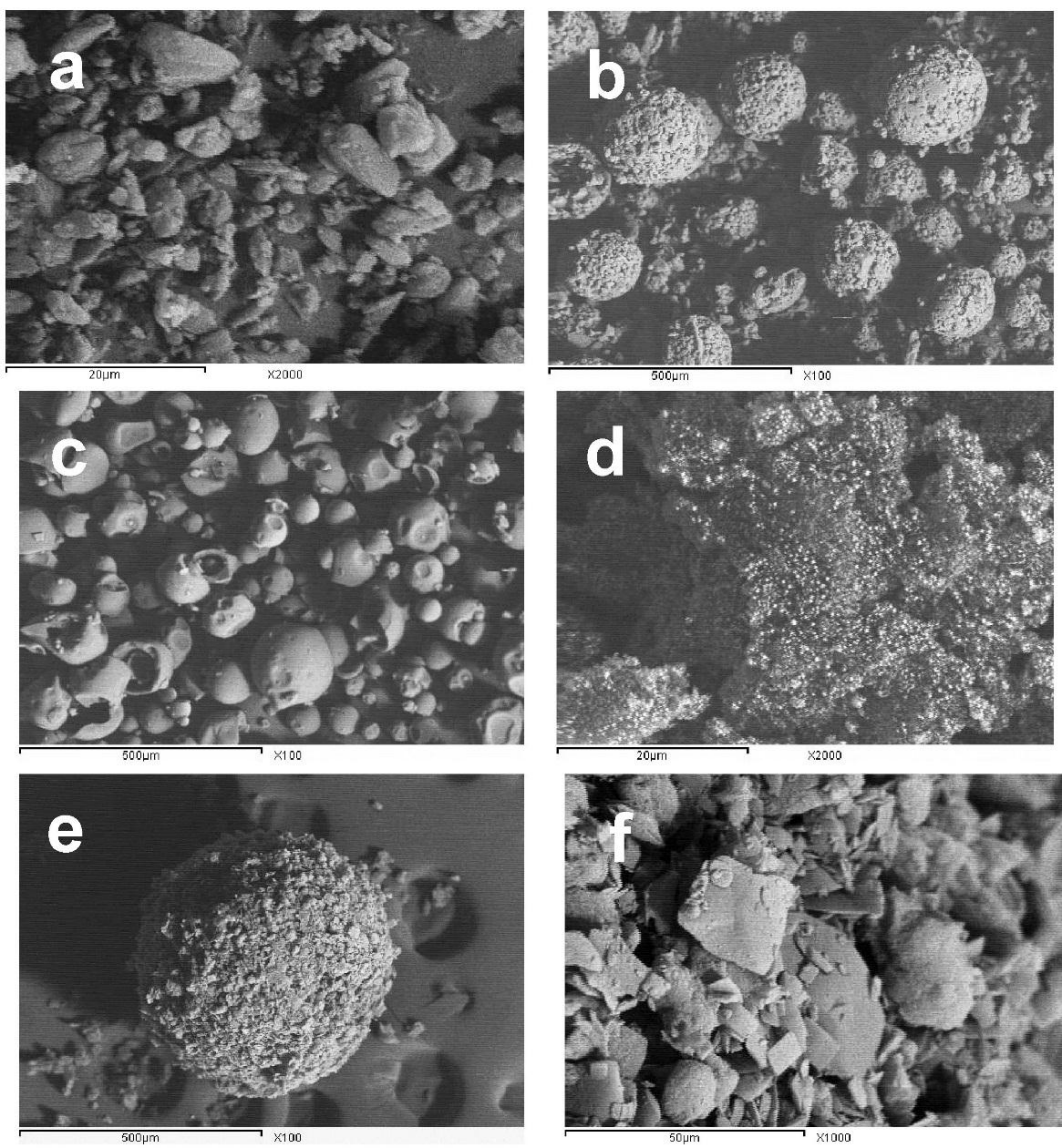
SEM micrographs of (a) pure glimepiride; (b) Starlac at 100 x; (c) PVP K30 at 100x; (d) Aerosil 200 at 2000x (e) spherical agglomerates with PVP & starlac [SAPVPst] whole spheres at 100x; (f) SAPVPst surface at 10000x

Figure 2 illustrates the elements used in the preparation of SA. Starlac particles were globular in shape with an irregular surface similar to lactose globules (which constitutes the larger percentage of such carrier). PVP K30 appeared as large smooth spheres while Aerosil 200 appeared as fine particles. The prepared SAPVPst agglomerates were much larger in size compared with the single components, perfectly spherical with a distinct rough surface. Higher magnification of agglomerate surfaces showed the aggregation of drug platelets together with occasional small spherical patches that might be due to the surface adsorption of Starlac particles.

### Optimization of tablet formulations prepared with TSDads & SA

Quality control tests for the prepared tablets showed acceptable results within the US Pharmacopeial limits (data not shown).

#### Disintegration time

Initial disintegration time (I.D.T) was thought to be the rate-limiting step in drug dissolution; therefore, it was mainly considered in tablet optimization. 

Formulae T1-T4 (Table 1) containing PreGelSt as an externally added superdisintegrant showed variation in I.D.T. The best value was recorded in T4 (1.51 min.) (Table 2). The high concentration of PreGelSt present in T4 might have added value to the swelling properties of the starch, helping the rapid uptake of water into the tablet core and causing its rapid disintegration. Tablets containing CP in T5 had the least value for I.D.T. (1.48 min). These results confirmed the superiority of CP over all tested superdisintegrants.^52-54^ Its unique porous structure along with its high hydration capacity^55^ resulted in a high swelling volume and an increase in the internally applied pressure inside tablet matrices. Thus, the rapid disintegration of tablets occurred at a much higher rate.

Tablets containing CP in TS2 (Table 2) also had the least value for D.T. (0.5 min), confirming its superiority.

Other superdisintegrants, either in TSDads or SA tablets, were ranked with respect to their efficiency in the following order Ac-Di-Sol> Starlac> Pearlitol flash. Although the marketed tablets Amaryl® showed spontaneous disintegration, yet the low release rate of its tablet overshadowed the good result for its D.T.

**Table 1:** Composition of triple solid dispersion adsorbate [TSDads] & spherical agglomerates [SA] tablets

**Formula Code**	**Superdisintegrant**		**Lubricant**	**Sweetener**	**Compression aid**	**Binder**	
	**Type**	**Weight (mg)**	**Mg Stearate(mg)**	**Aspartame (mg)**	**Pearlitol SD (mg)**	**Avicel (mg)**	**L-HPC (mg)**
T1*	PreGelSt^#^	25	2.5	5	25	32.9	6.6
T2*	PreGelSt^#^	25	0.625	5	25	34.47	6.89
T3*	PreGelSt^#^	50	0.625	5	25	13.64	2.72
T4*	PreGelSt^#^	50	0.625	5	20	15	5
T5*	CP^##^	50	0.625	5	20	15	5
T6*	Ac-Di-Sol	50	0.625	5	20	15	5
T7*	Pearlitol flash	50	0.625	5	20	15	5
T8*	Starlac	50	0.625	5	20	15	5
TS1**	PreGelSt^#^	50	0.625	5	20	15	5
TS2**	CP^##^	50	0.625	5	20	15	5
TS3**	Starlac	50	0.625	5	20	15	5
TS4**	Ac-Di-Sol	50	0.625	5	20	15	5
TS5**	Pearlitol flash	50	0.625	5	20	15	5

*All formulae contain 153 mg of the optimized TSDads equivalent to 3 mg glimepiride **All formulae contain 6.6 mg of the optimized SA equivalent to 3 mg glimepiride and 143.7 mg lactose as diluents # Pregelatinized starch ## Crosspovidone

**Table 2:** In-vitro disintegration time for tablet formulae containing triple solid dispersion adsorbate [TSDads] & spherical agglomerates [SA]

**Formula Code**	**Initial disintegration time I.D.T. (min.)**	**Total disintegration time T.D.T (min.)**
**Amaryl®**	0.25	2.00
T1*	3.01	16.20
T2*	2.56	14.12
T3*	2.30	10.50
T4*	1.51	8.42
T5*	1.48	6.29
T6*	2.07	9.16
T7*	3.53	9.39
T8*	3.11	8.12
TS1**	1.34	4.75
TS2**	0.50	1.09
TS3**	1.02	3.24
TS4**	0.86	3.19
TS5**	1.02	2.41

* Triple solid dispersion adsorbate TSDads tablets ** Spherical agglomerates SA tablets

#### Kinetic analysis of release data 

Kinetic treatment of glimepiride release data (Table 3) showed that a diffusion model prevailed in most of the TSDads tablets except for T6 (with Ac-Di-Sol), where a first order release and a small percentage of flush release occurred. The other formulae demonstrated different lag time values. This variation might be a result of a difference in the wetting capability within the tablet core. T1 possessed the longest lag time (7.9 min) While proceeding in optimization, lag time values decreased sequentially with the successive decrease in binder weights along with the increase in the amount of added PreGelSt.^56,57^ It seemed that the concomitant variation in these two excipients was a promising factor that predisposes the particles earlier to the wetting effect of the dissolution medium. The least value was attained in T5 (1.01 min) containing CP. This proved its superiority in achieving the highest rate of wetting to tablet matrix before the release began to proceed.

**Table 3:** Kinetic treatment of release data of glimepiride from triple solid dispersion adsorbate [TSDads] & spherical agglomerates [SA] tablets

**Formula Code**	**Order of release**	**K** ^***^	**Half- life (min.)**	**Y-intercept**	**Significance of Y-intercept**	
					**Flush release (mg%)**	**Lag time (min.)**
T1*	diffusion	8.75	72.62	-24.63	-	7.92
T2*	diffusion	9.12	66.90	-24.67	-	7.31
T3*	diffusion	9.78	59.70	-25.71	-	6.90
T4*	diffusion	8.03	60.82	-12.62	-	2.47
T5*	diffusion	7.86	54.13	-7.93	-	1.02
T6*	first	0.01	61.43	1.99	1.67	-
T7*	diffusion	7.45	66.27	-10.84	-	2.12
T8*	diffusion	8.33	57.50	-13.25	-	2.53
TS1**	diffusion	6.74	75.17	-8.44	-	1.57
TS2**	diffusion	7.90	59.83	-11.11	-	1.98
TS3**	diffusion	7.29	72.35	-12.01	-	2.72
TS4**	first	0.01	64.02	1.98	4.50	-
TS5**	first	9.902x10^-3^	69.98	1.97	5.16	-
**Amaryl®**	zero	0.37	122.98	4.02	4.03	-

* triple solid dispersion adsorbate TSDads Tablets, ** spherical agglomerates SA tablets ***Units of K ( rate constant) is mg/min for zero order, min^-1^ for first order & mg/ min^1/2^for Higushi diffusion model

Tested superdisintegrants in SA tablets acted differently within their respective matrices. Tablets with Ac-di-sol (TS4) and Pearlitol flash (TS5) showed a similar behavior, where a first order kinetics prevailed with a similar magnitude of flush release. On the contrary, release from tablets containing PreGelSt (TS1), CP (TS2) and Starlac (TS3) matched with a perfect diffusion model with different lag time values. All SA tablets showed variable release rates. This variation might be a result of a difference in the wetting capability within their tablet cores. Different types of added superdisintegrants contributed to that difference. The tablet formula (TS2) containing CP was considered optimum, as it showed the least release t_1/2_ (59.8 min), as well as an acceptable short lag time value (1.98 min).

#### Physicochemical characterization of optimized tablet formulae containing TSDads and SA

#### Scanning Electron Microscopy (SEM) 

**Figure 3:**
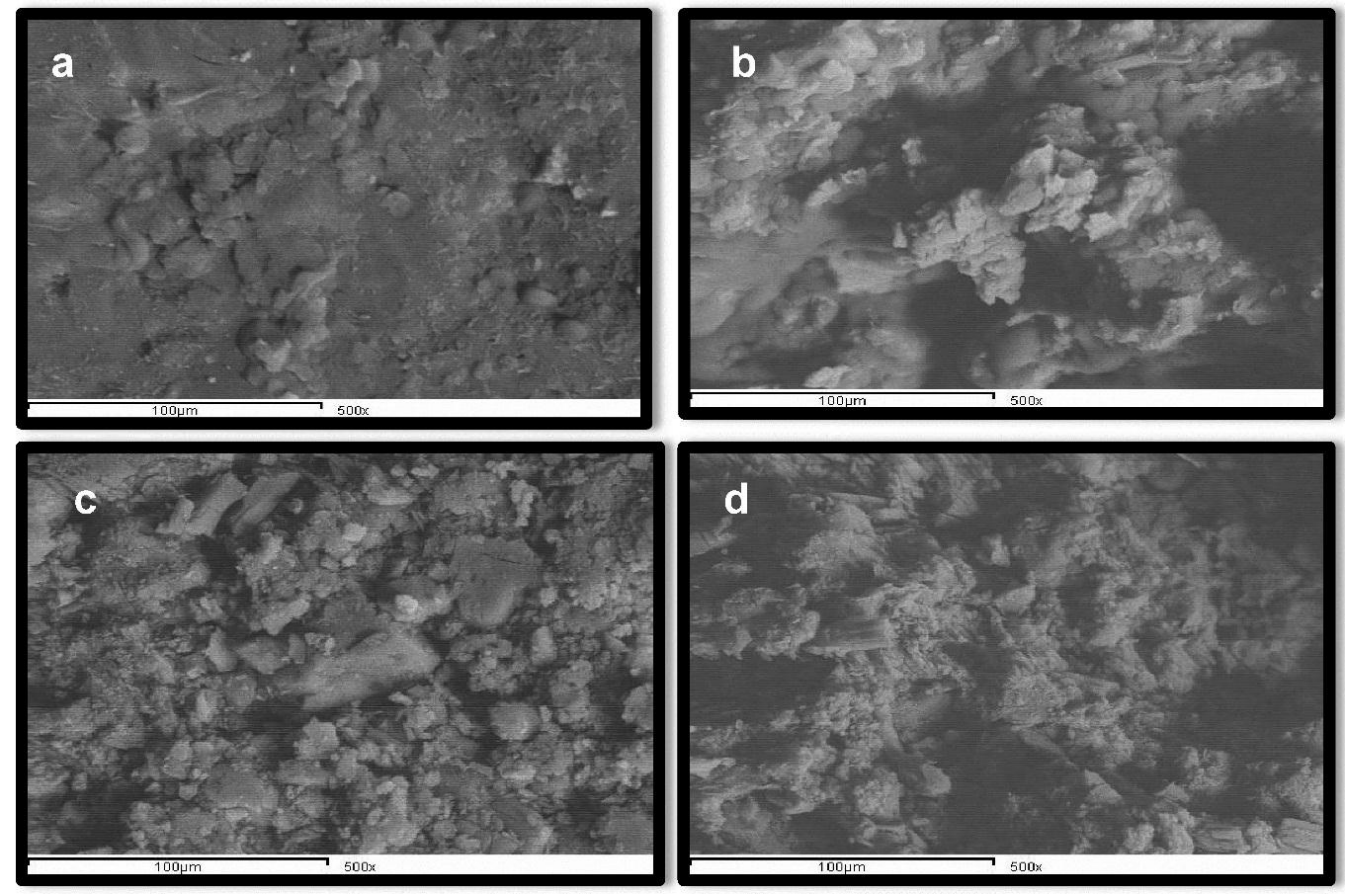
SEM for optimized triple solid dispersion adsorbate [TSDads] tablet formula (T5) (a) Surface view, (b) Cross section view; optimized spherical agglomerates [SA] tablet formula (TS2) (c) Surface view, (d) Cross section view

A surface view of tablets containing TSDads showed a rough non-planar surface with occasional protrusions (Figure 3a). Occasional pores were clearly identified at a higher magnification power (1500x). The pores were extending to the interior of the core structure, as illustrated in the cross-sectional view (Figure 3b).Tablets containing SA showed a more extensive rough reticulated surface with more frequent pores extending to the tablet core (Figure 3c,3d). Spherical crystals of the drug might account for the obvious reticulation on the surface of their respective tablets. As clearly demonstrated, tablets with either TSDads or SA with CP as an external superdisintegrant gave upon compression a perfect design for a well-organized interconnected porous matrix. This was confirmed by the kinetic treatment of the release data in which glimepiride release from such matrices obeyed Higuchi diffusion model (Table 3).

#### Effect of compression on glimepiride release 

**Figure 4:**
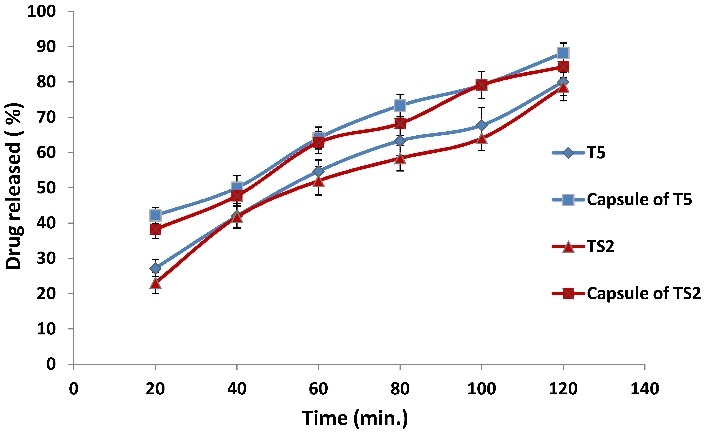
Comparison of release profile of glimepiride from capsules containing the constituents of T5 or TS2 to their respective tablets T5 or TS2

An important reason which prevents the scaling up of both solid dispersion and spherical crystal techniques industrially was the fragility of their matrices and the high probability of destruction upon compression. That is why tablets were compressed at a low compression force, and the effect of compression on release was depicted. Results shown in Figure 4 illustrate similar release rates for capsules ofT5, TS2 and their respective tablets as indicated by the nearly parallel curves in either case. A high value for similarity factor (Table 4) confirmed the results in both cases. Also, a similar extent of release after 120 min. was demonstrated, where T5 gave 88% and 80% release for capsules and tablets, respectively, & TS2 gave 84% and 79% release, respectively, before and after tabletting. This result confirmed the success of glimepiride tablet formulation to provide high extent of drug release by either technique adopted, and it can be postulated that the low compression force applied protected the integrity of the solid dispersion and spherical crystals upon tabletting, offering a great opportunity for the success of both techniques on industrial scale production.

Kinetic data of release for both formulae before and after compression (Table 4) revealed a common release mechanism in capsule form for T5 and TS2 (zero order), which was shifted to a diffusion model in the tablet form. Furthermore, capsules of T5 and TS2 showed a similar flush release which, upon tabletting, turned to similar lag times values. As stated before, an organized matrix structure was illustrated in tablet form (Figure 3b, d) from which the diffusion-controlled release predominated. The time necessary for the dissolution medium to access drug particles inside respective matrices accounted for the encountered lag time.

**Table 4:** Kinetic treatment of release data of glimepiride from optimized triple solid dispersion adsorbate [TSDads] & spherical agglomerates [SA] before and after compression

**Formula Code**		**Order of release**	K****	**Similarity Factor f**_2_ ***	**Half-life (min.)**	**Y-intercept**	**Significance of Y-intercept**	
							**Flush release (%)**	**Lag time (min.)**
T5*	**Tablet**	diffusion	7.86	99.668	54.13	-7.93	-	1.02
	**Capsule**	zero	0.47		36.79	32.65	32.65	-
TS2**	**Tablet**	diffusion	7.90	98.837	59.83	-11.11	-	1.98
	**Capsule**	zero	0.47		43.69	29.07	29.07	-

*TSDads, **SA
$$***f_{2}=50.log\left[\frac{100}{\sqrt{1+\sum^{\text{​}}\frac{{\left(capsule-tablet\right)}^{2}}{n}}}\right]$$
****Units of K ( rate constant) is mg/min for zero order, min-1 for first order & mg/ min1/2 for Higushi diffusion model

The similarity in results between the two optimized formulae based on either TSDads or SA might rely upon the same type and percentage of the added superdisintegrant. CP acted upon the formulae in capsule form through a strong wetting and swelling action. It acted also on both tablet formulae by creating similar interconnecting channels from which a similar release rate was shown (Table 4).

#### Pharmacodynamic evaluation of optimized tablet formulae

**Figure 5:**
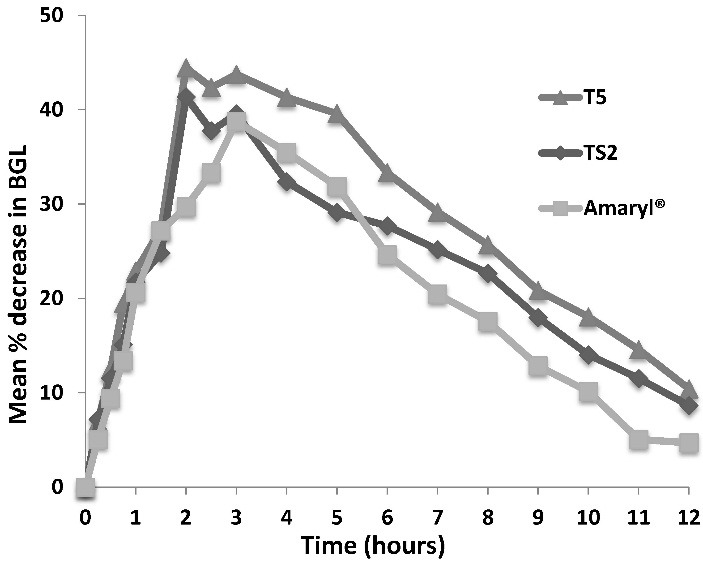
Mean percent decrease in blood glucose level [BGL] of normal rabbits receiving Amaryl®, formula TS2 and formula T5

The mean percent reduction in blood glucose level (BGL) for the treated rabbits versus time after administration of the marketed product Amaryl®, formula TS2 and formula T5 is represented in Figure 5. Maximum percent reduction in BGL (Red max), the corresponding time (Tmax) and the Area under the Curve (AUC _0-12_) were calculated using *Kinetica*® software.

Both tablet formulae gave higher values for Red max and Tmax was attained earlier than that of the marketed product. Therefore, the two new tablet formulations were thought to be more efficient in their hypoglycemic effect, as illustrated in Table 5 & Figure 5. Results were then analyzed statistically using the one-way analysis of variance (*ANOVA*) to determine the least significant difference, if any, between the tested formulae and the marketed product. The difference between formula T5 and TS2 in Red max, AUC_0-12_ and Tmax was found to be non-significant (*p*>0.05), suggesting an equivalent therapeutic efficacy for either tested formula. However, there was a significant difference between the value of Tmax of the tablet formula and that of the marketed product (*p*<0.05). This could support the goal of our work in which the enhancement in glimepiride dissolution through tablet formulation had contributed to a more rapid onset of action, which could be of value in acute cases of hyperglycemia.

**Table 5:** Comparison between pharmacokinetic parameters of optimized tablets with marketed product

**Pharmacokinetic parameters**	**Amaryl®**	**TS2**	**T5**
Red max (maximum % decrease in BGL*) ± S.D.	40.07±10.14	42.89±4.49	48.58±3.84
Tmax ( time to attain maximum % decrease in BGL) ± S.D.	2.87±0.25	2.12±0.25	2.50±0.57
AUC _0-12_ ± S.D.	244.07±56.02	277.34±72.55	328.43±118.73

**S.D.:** Standard Deviation. * BGL: Blood glucose level

## Conclusion

The inclusion of glimepiride in a matrix of either triple solid dispersion adsorbates or spherical agglomerates appeared to be equally successful in achieving the target of experimental work. An extensive enhancement in glimepiride release from such formulae occurred, accounting for an average t_1/2_ less than 60 min, while that of the marketed product extended to about 123 min. Furthermore, an *in vivo* hastening in the onset time occurred, where the hypoglycemic effect appeared about 2h after the oral administration of either formula to male albino rabbits relative to 3h in case of the marketed product. Hence, the results of this study demonstrate the potential of either studied techniques in enhancing both the *in vitro* and *in vivo* performance of glimepiride through oral tablet formulation.

## Ethical Issues

Not applicable

## Conflict of Interest

The authors declare no conflict of interests.
